# The linker domain of the SNARE protein SNAP25 acts as a flexible molecular spacer that ensures efficient S-acylation

**DOI:** 10.1074/jbc.RA120.012726

**Published:** 2020-04-21

**Authors:** Christine Salaun, Jennifer Greaves, Nicholas C. O. Tomkinson, Luke H. Chamberlain

**Affiliations:** ‡ Strathclyde Institute of Pharmacy and Biomedical Sciences, University of Strathclyde, Glasgow G4 0RE, United Kingdom; § Faculty of Health and Life Sciences, Centre for Sport, Exercise and Life Sciences, Coventry University, Coventry CV1 5FB, United Kingdom; ¶ WestCHEM, Department of Pure and Applied Chemistry, University of Strathclyde, Glasgow G1 1XL, United Kingdom

**Keywords:** protein palmitoylation, post-translational modification (PTM), protein acylation, protein chemical modification, protein domain, ankyrin repeat domain, mPEG-click, S-acylation, zDHHC enzyme, zDHHC17

## Abstract

*S*-Acylation of the SNARE protein SNAP25 (synaptosome-associated protein of 25 kDa) is mediated by a subset of Golgi zinc finger DHHC-type palmitoyltransferase (zDHHC) enzymes, particularly zDHHC17. The ankyrin repeat domain of zDHHC17 interacts with a short linear motif known as the zDHHC ankyrin repeat–binding motif (zDABM) in SNAP25 (^112^VVASQP^117^), which is downstream of its *S*-acylated, cysteine-rich domain (^85^CGLCVCPC^92^). Here, we investigated the importance of a flexible linker region (amino acids 93–111, referred to hereafter as the “mini-linker” region) that separates the zDABM and *S*-acylated cysteines in SNAP25. Shortening the mini-linker did not affect the SNAP25–zDHHC17 interaction but blocked *S*-acylation. Insertion of additional flexible glycine-serine repeats had no effect on *S*-acylation, but extended and rigid alanine-proline repeats perturbed it. A SNAP25 mutant in which the mini-linker region was substituted with a flexible glycine-serine linker of the same length underwent efficient *S*-acylation. Furthermore, this mutant displayed the same intracellular localization as WT SNAP25, indicating that the amino acid composition of the mini-linker is not important for SNAP25 localization. Using the results of previous peptide array experiments, we generated a SNAP25 mutant predicted to have a higher-affinity zDABM. This mutant interacted with zDHHC17 more strongly but was *S*-acylated with reduced efficiency in HEK293T cells, implying that a lower-affinity interaction of the SNAP25 zDABM with zDHHC17 is optimal for *S*-acylation efficiency. These results show that amino acids 93–111 in SNAP25 act as a flexible molecular spacer that ensures efficient coupling of the SNAP25–zDHHC17 interaction and *S*-acylation of SNAP25.

## Introduction

*S*-Acylation (also called palmitoylation), the reversible attachment of fatty acids onto cysteine residues, occurs on a wide range of eukaryotic proteins (1). This post-translational modification regulates membrane interactions of soluble proteins and mediates stabilization and trafficking of both soluble and transmembrane proteins (2). The occurrence of *S*-acylation is widespread (3, 4), with synaptic proteins apparently over-represented in the *S*-acylated proteome: 41% of synaptic proteins were suggested to be modified by *S*-acyl chains (5). Many of the *S*-acylated synaptic proteins that have been characterized are reversibly modified and undergo *S*-acylation cycling mediated by zDHHC^3^ *S*-acyltransferase and APT/ABHD thioesterase enzymes localized at synaptic regions (5–9). However, initial *S*-acylation of newly synthesized synaptically targeted proteins is likely to occur in the cell body, mediated by zDHHC enzymes localized to endoplasmic reticulum or Golgi compartments (8).

There are 23 zDHHC enzyme isoforms in the human genome (6). The encoded enzymes are transmembrane proteins that are predominantly associated with endoplasmic reticulum and Golgi compartments (10). The catalytic domain of zDHHC enzymes is a 51-amino acid cysteine-rich domain containing an aspartate-histidine-histidine-cysteine (DHHC) motif. The *S*-acylation reaction occurs via a two-step process whereby the cysteine of the DHHC motif undergoes autoacylation prior to transfer of the acyl chain to substrate cysteine (11, 12). The catalytic cysteine of zDHHC enzymes is positioned at the cytosolic face of the membrane (13, 14), and therefore substrate cysteines must be at a similar position to allow *S*-acylation. Although palmitic acid is the main fatty acid used in *S*-acylation reactions, zDHHC enzymes can also use shorter and longer chain fatty acids as substrates (15). The fatty acid selectivity of these enzymes is determined by specific amino acids in the transmembrane domains that determine the length of acyl chain that can be accommodated within the tepee-like cavity formed by the transmembrane helices (11, 14, 16).

Although zDHHC enzymes were recognized as *S*-acyltransferases over 15 years ago (6), there is very little information available about the substrate networks of individual enzymes and how enzyme-substrate specificity is encoded. Some zDHHC enzymes, such as zDHHC3 and zDHHC7, appear to exhibit very loose substrate specificity and may have minimal interactions with their substrates (17). Instead these enzymes may rely on a very high intrinsic activity to mediate *S*-acylation of any accessible cysteines at the membrane interface (17). In contrast, some other zDHHC enzymes exhibit a much more restricted substrate network. zDHHC17 and zDHHC13 are unique in the zDHHC family because they contain an N-terminal ankyrin-repeat (ANK) domain. ANK domains are present in many different proteins and are recognized as protein interaction modules. The ANK domain of zDHHC17 is essential for *S*-acylation of substrates such as huntingtin, SNAP25 (synaptosomal-associated protein of 25 kDa), and CSP (cysteine-string protein) (17, 18). Our previous work identified a consensus recognition short linear motif in these and other substrates that mediate binding to the ANK domain of both zDHHC17 and zDHHC13 (19). The six-amino acid [VIAP][VIT]*XX*QP consensus motif makes key contacts with asparagine 100 and tryptophan 130 of zDHHC17 (20). We named the consensus short linear motif the zDHHC ankyrin repeat–binding motif (zDABM) (21). The affinity of the SNAP25 zDABM for the ANK domain of zDHHC17 is ∼11 μm, although full-length SNAP25 has a higher affinity (0.5 μm) (20), likely because of optimization of binding/presentation of the zDABM to zDHHC17. The presence of other zDHHC17-binding sites in SNAP25 is unlikely because mutation of proline 117 in the zDABM blocks binding to the ANK domain, *S*-acylation, and membrane targeting (19, 22, 23).

Using peptide arrays, we defined the sequence rules of the zDABM of SNAP25 and CSP and used these rules to predict and validate a number of new zDHHC17 interactors (21). This analysis suggested that different zDABMs have different affinities for the ANK domain of zDHHC17 and that this can also be influenced by the identity of nonconserved residues (*i.e.* the *X* position in the zDABM) or surrounding amino acids (21). Furthermore, using the generated sequence rules of interaction, it was possible to create zDABM sequences that displayed increased interactions with the ANK domain of zDHHC17 (21). An interesting observation that came from this analysis is that not all of proteins that contain zDABMs are *S*-acylated by zDHHC17 (21), suggesting that other features of the binding partner must dictate whether or not it is an effective *S*-acylation substrate. Although the crystal structure of the ANK domain of zDHHC17 (with and without substrate peptide) has been reported (20), there is currently no structural information available for the full-length enzyme. Thus, it is unclear how the substrate-binding ANK domain and the catalytic DHHC domain of zDHHC17 are positioned relative to each other. Unlike other zDHHC enzymes, zDHHC17 and zDHHC13 are predicted to contain six (rather than four) transmembrane domains (24), and it is likely that the additional two transmembrane helices play some role in mediating the correct positioning of the ANK domain relative to the bilayer and DHHC domain.

Our hypothesis is that substrates of zDHHC17 require both a zDABM and also accessible cysteine residues that are appropriately positioned to allow simultaneous engagement of the zDABM with the ANK domain and the cysteines with the DHHC domain. Thus, the relative orientations and spatial separation of the ANK domain and catalytic DHHC domain of zDHHC17 must be mirrored in the zDABM and cysteines of substrate proteins to allow effective *S*-acylation. We also propose that the affinity of the zDABM–ANK domain interaction is suitably optimized to allow both efficient substrate engagement *and* release following *S*-acylation. In this study, we have tested these ideas by modifying the “mini-linker” region of SNAP25 that connects the zDABM and *S*-acylated cysteines. The results suggest that the sequence of the linker is not important for efficient *S*-acylation by zDHHC17 but that linker length and flexibility are important features for *S*-acylation. In addition, we have used knowledge about the zDABM–ANK domain interaction to generate a SNAP25 mutant that displays enhanced binding to zDHHC17. This mutant displays reduced *S*-acylation by zDHHC17 in HEK293T cells, demonstrating the importance of binding affinity for *S*-acylation efficiency.

## Results

### Shortening the mini-linker region between the S-acylation domain and the zDABM of SNAP25 has no effect on zDHHC17 interaction but blocks S-acylation

**Figure 1.**
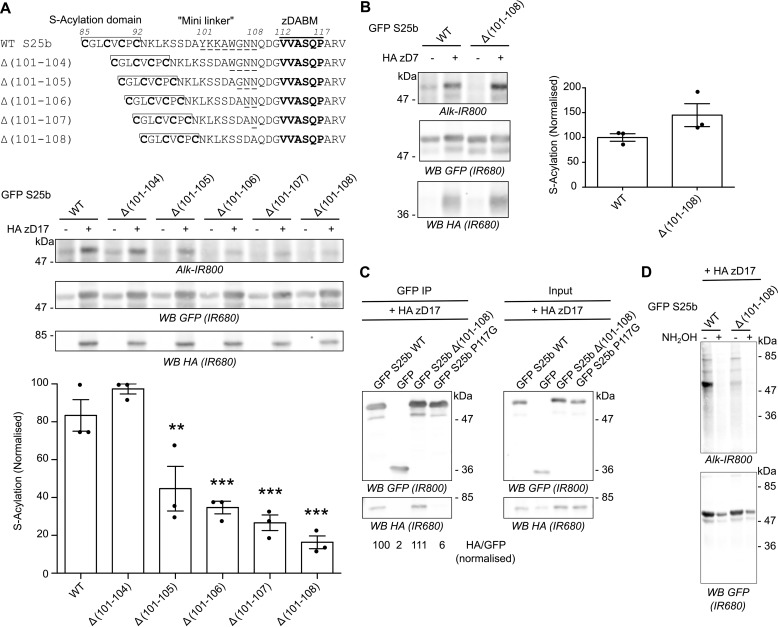
**Shortening the linker region of SNAP25 decreases *S*-acylation by zDHHC17 without impairing interaction with the enzyme.** *A*, *top panel*, schematic of the deletions introduced in the linker region of EGFP-SNAP25. *Middle* and *bottom panels*, HEK293T cells were transfected with either EGFP SNAP25b WT (*GFP S25b WT*) or the various deletion mutants (Δ(101–104), Δ(101–105), Δ(101–106), Δ(101–107), or Δ(101–108)), together with either a plasmid encoding HA zDHHC17 (+ *HA zD17*) or a control pEF-BOS HA plasmid (− *HA zD17*). The cells were then incubated with 100 μm C16:0-azide, and the proteins incorporating the azide fatty acid were labeled by click chemistry using an alkyne-IR 800 dye (*Alk-IR800*). The proteins were resolved by SDS-PAGE and transferred to nitrocellulose membranes. Representative images are shown (*middle panel*): click chemistry signal (*top row*, *Alk IR800*), GFP (*middle row*, *IR680*), and HA (*bottom row*, *IR680*) immunoblots. The *graph* (*bottom panel*) shows means ± S.E. of normalized *S*-acylation with HA zDHHC17; *filled circles* represent individual samples (*n* = 3 different cell samples for each condition). Statistical analysis (ANOVA) showed no significant difference between the zDHHC17 mediated *S*-acylation of WT *versus* Δ(101–104), whereas there was a significant difference for WT *versus* Δ(101–105), Δ(101–106), Δ(101–107), or Δ(101–108). **, *p* < 0.01; ***, *p* < 0.001. *B*, HEK293T cells were transfected with EGFP SNAP25b WT (*GFP S25b WT*), the Δ(101–108) deletion mutant together with HA zDHHC7 (+ *HA zD7*), or a control pEF-BOS HA plasmid (− *HA zD7*). The cells were then treated as in *A*. Representative images are shown in the *left column*: click chemistry signal (*top row*, *IR 800*), GFP (*middle row*, *IR 680*), and HA (*bottom row*, *IR680*) immunoblots. The *graph* on the *right* shows means ± S.E. of normalized *S*-acylation with HA zDHHC7; *filled circles* represent individual samples (*n* = 3 different cell samples for each condition). Statistical analysis (Student's *t* test) showed no significant difference between the *S*-acylation of WT *versus* Δ(101–108) by zDHHC7. *C*, HEK293T cells were transfected with EGFP SNAP25b WT (*GFP S25b WT*), the Δ(101–108) deletion mutant (*GFP S25b* Δ*(101–108)*), or additional control plasmids (*GFP SNAP25b P117G* or *GFP*) together with HA zDHHC17 (+ *HA zD17*). Lysates (*right panel*, *Input*) were immunoprecipitated (*IP*) with an anti-GFP antibody (*left panel*), resolved by SDS-PAGE, and transferred to nitrocellulose membranes. The membranes were probed with anti-GFP (*top panel*) or anti-HA (*bottom panel*) antibodies. The ratio between the HA signal and the GFP signal was quantified for the immunoprecipitated samples and is indicated at the *bottom. D*, HEK293T cells were transfected with either EGFP SNAP25b WT (GFP S25b) or the Δ(101–108) deletion mutant (GFP S25b Δ(101–108)) together with a plasmid encoding HA zDHHC17 (+*HA zD17*) and were treated as in *A*. Click-chemistry labeled samples were then concentrated by acetone precipitation, aliquoted, and incubated with an equal volume of either 2 m hydroxylamine (+ *NH_2_OH*) or 2 m Tris (− *NH_2_OH*) for 1 h at room temperature. They were then resolved and analyzed by SDS-PAGE. The *top panel* shows the click chemistry signal (*Alk-IR800*), whereas the *bottom panel* shows the anti-GFP immunoblot (*IR680*). The positions of molecular mass markers (in kDa) are shown on the *right* or *left side* of all immunoblots. *WB*, Western blot.

The linker domain of SNAP25 is the region of the protein that separates the two SNARE domains (25). This ∼60-amino acid region includes the *S*-acylated cysteines (amino acids 85–92) and the zDABM (amino acids 112–117). In this study, we were interested in the region of SNAP25 separating the cysteine-rich domain and zDABM (*i.e.* residues 93–111) and refer to this region herein as the mini-linker (Fig. 1*A*).

In previous work, we reported that shortening the mini-linker of SNAP25 led to a loss of membrane association induced by zDHHC17 co-expression (23). To investigate this further, we examined *S*-acylation of a range of SNAP25 mutants with deletions of between four and eight amino acids from the mini-linker. *S*-Acylation was assessed by incubating transfected cells in C16:0-azide, and proteins incorporating the azide were subsequently labeled using click chemistry with an alkyne IR dye. As shown in Fig. 1*A*, there was a marked and significant loss of *S*-acylation when five or more amino acids were removed, with deletion of eight amino acids having the greatest effect. This loss of *S*-acylation was specific to zDHHC17 and was not observed with the high activity/low selectivity enzyme, zDHHC7 (Fig. 1*B*). These results are consistent with our previous demonstration of a loss of membrane binding of these mutants when co-expressed with zDHHC17 (23). To examine whether loss of *S*-acylation was associated with loss of binding to zDHHC17, we undertook immunoprecipitation (IP) experiments (Fig. 1*C*). As shown, IP of EGFP-SNAP25 led to co-IP of HA-zDHHC17. A similar result was seen with the SNAP25 Δ(101–108) mutant, suggesting that deletion of these residues from the mini-linker has no effect on interaction of SNAP25 with zDHHC17 in HEK293T cells. In contrast, substitution of proline 117 in the zDABM of SNAP25 with a glycine residue led to a complete loss of co-IP of HA-zDHHC17, which demonstrates that this approach faithfully reports on interactions of zDHHC17 with the zDABM of SNAP25 (proline 117 is essential for this interaction) (19). To confirm that the C16:0-azide was attached to cysteine residues in both the WT and Δ(101–108) mutant, cell lysates were treated without or with 1 m hydroxylamine, which cleaves thioesters; although this treatment led to some loss of immunoreactivity, there was clearly a loss of click signal (Fig. 1*D*), consistent with C16:0-azide on both proteins being linked by hydroxylamine-sensitive thioester bonds.

### mPEG-Click reveals the number of S-acylated cysteines on SNAP25 proteins

**Figure 2.**
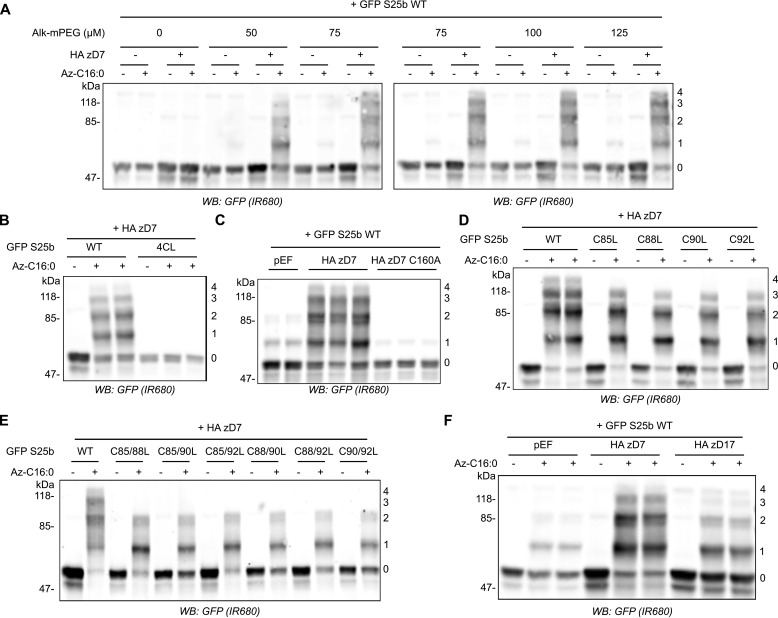
**The mPEG-Click technique enables detection of the number of *S*-acylated cysteines on SNAP25.** *A*, HEK293T cells were transfected with EGFP SNAP25b WT (*GFP S25b WT*) together with a plasmid encoding HA zDHHC7 (+ *HA zD7*) or a control pEF-BOS HA plasmid (− *HA zD7*). The cells were then incubated with 100 μm C16:0-azide (+ *Az C16:0*) or palmitic acid (− *Az C16:0*). Proteins incorporating C16:0-azide were labeled by click chemistry using increasing concentrations of alkyne-mPEG 5K (0–125 μm). Proteins were resolved by SDS-PAGE, transferred to nitrocellulose membranes, and probed with a GFP antibody. Molecular mass markers are shown on the *left*, whereas the *numbers* on the *right* relate to the number of modified cysteines detected. *B*, HEK293T cells were transfected with either EGFP SNAP25b WT or an EGFP SNAP25b mutant in which all cysteines had been mutated to leucines (*4CL*) together with a plasmid encoding HA zDHHC7 (+ *HA zD7*). The cells were then incubated with 100 μm C16:0-azide (+ *Az C16:0*) or palmitic acid (− *Az C16:0*). Proteins incorporating C16:0-azide were labeled by click chemistry using 100 μm alkyne-mPEG 5K. Isolated proteins were resolved by SDS-PAGE, transferred to nitrocellulose membranes, and probed with a GFP antibody. *C*, HEK293T cells were transfected with EGFP SNAP25b WT (*GFP S25b WT*) together with a plasmid encoding HA zDHHC7 (+ *HA zD7*), an inactive mutant of zDHHC7 in which the catalytic cysteine had been mutated into an alanine (*HA zD7 C160A*), or control pEF-BOS HA plasmid (*pEF*). The cells were processed as described for *B. D*, HEK293T cells were transfected with EGFP SNAP25b WT or single cysteine mutants (*C85L*, *C88L*, *C90L*, and *C92L*) together with a plasmid encoding HA zDHHC7 (+ *HA zD7*). The cells were then labeled and analyzed as in *B. E*, HEK293T cells were transfected with EGFP SNAP25b WT or double cysteine mutants (*C85/88L*, *C85/90L*, *C85/92L*, *C88/90L*, *C88/92L*, and *C90/92L*) together with a plasmid encoding HA zDHHC7 (+ HA zD7). The cells were then labeled and analyzed as in *B. F*, HEK293T cells were transfected with EGFP SNAP25b WT together with a plasmid encoding HA zDHHC7 (*HA zD7*), HA zDHHC17 (*HA zD17*), or a control plasmid pEF-BOS HA (*pEF*). The cells were then labeled and analyzed as in *B. WB*, Western blot.

SNAP25 has four potential *S*-acylated cysteines. The reduction in click chemistry signal using alkyne IR dye (Fig. 1) could therefore reflect any of the following: (i) a reduction in the total number of SNAP25 molecules that are *S*-acylated; (ii) a reduction in the number of cysteines that are modified on individual SNAP25 molecules; or (iii) a combination of (i) and (ii). We therefore sought to implement a different click chemistry labeling approach that would allow these possibilities to be discriminated. For this, instead of reacting C16:0-azide with an alkyne IR dye, we reacted it with a 5-kDa PEG Alkyne (Alk-mPEG) and characterized this methodology using cells transfected with EGFP-SNAP25 and HA-zDHHC7. As can be seen in Fig. 2*A*, this approach allowed visualization of four distinct PEGylated bands for SNAP25 (labeled *1–4*). To confirm that these immunoreactive bands represent SNAP25 modified by 1–4 mPEG groups (and hence SNAP25 *S*-acylated on 1–4 cysteines), we showed that: (i) these PEGylated bands were lost when all four cysteines in SNAP25 were mutated to leucines (Fig. 2*B*); (ii) PEGylated bands were not observed with a catalytically dead zDHHC7(C160A) mutant (Fig. 2*C*); and (iii) there was a corresponding loss of the upper PEGylated band when one cysteine was mutated and a loss of the upper two PEGylated bands when two cysteines were mutated in SNAP25 (Fig. 2, *D* and *E*). As well as providing a methodology to more thoroughly investigate *S*-acylation of SNAP25, to our knowledge, this analysis also provides the first *direct* evidence that SNAP25 can be modified simultaneously on all four of its cysteines. Finally, we compared the *S*-acylation profile of SNAP25 when co-expressed with DHHC7 *versus* DHHC17 (Fig. 2*F*). The WT SNAP25 protein appeared to be modified differently by zDHHC7 and zDHHC17; the amount of SNAP25 modified with two or three mPEG groups was lower with zDHHC17 than with zDHHC7. mPEG-Click therefore reveals that SNAP25 is less heavily *S*-acylated by zDHHC17 than by zDHHC7, which is consistent with zDHHC17 being a low-activity/high-selectivity enzyme and zDHHC7 a high-activity/low-selectivity enzyme (17). Although a higher-molecular-mass SNAP25 band was present with zDHHC17 co-expression that migrated at the same size as SNAP25 modified by four mPEG groups, this band was present in both the absence and presence of C16:0-azide labeling (compare − and + Az-C16:0 samples) and thus is not a PEGylated form of the protein. Instead, we believe that this is an aggregated form of SNAP25 that arises because of incomplete *S*-acylation by zDHHC17.

**Figure 3.**
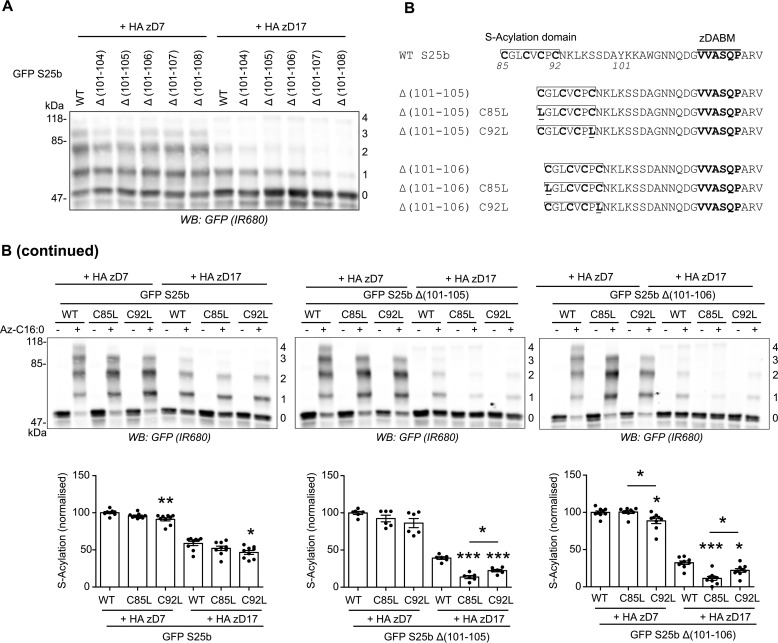
**mPEG-Click analysis of SNAP25 linker deletion mutants.** *A*, HEK293T cells were transfected with either EGFP SNAP25b WT (*GFP S25b WT*) or the various deletion mutants (Δ(101–104), Δ(101–105), Δ(101–106), Δ(101–107), or Δ(101–108)) together with a plasmid encoding either HA zDHHC7 (+ *HA zD7*) or HA zDHHC17 (+ *HA zD17*). The cells were then incubated with C16:0 azide, and proteins incorporating the fatty acid azide were labeled by click chemistry using alkyne-mPEG 5K. Proteins were resolved by SDS-PAGE, transferred to nitrocellulose membranes, and probed with a GFP antibody. The positions of molecular mass markers are shown on the *left*, whereas the *numbers* on the *right* relate to the number of modified cysteines in SNAP25. *B*, schematic of the mutants of EGFP SNAP25b used in the analysis. Cysteine-to-leucine mutations (*underlined*) were introduced at position 85 (*C85L*) or 92 (*C92L*) of EGFP SNAP25b WT (*WT S25b*) or the deletion mutants EGFP SNAP25b Δ(101–105) and Δ(101–106). *B (continued)*, HEK293T cells were transfected with the various constructs described above together with a plasmid encoding either HA zDHHC7 (*HA zD7*) or HA zDHHC17 (*HA zD17*). The cells were then incubated with C16:0 azide (+ *Az C16:0*) or palmitic acid (− *Az C16:0*). Proteins incorporating the fatty acid azide were labeled by click chemistry using alkyne-mPEG 5K. Isolated proteins were resolved by SDS-PAGE, transferred to nitrocellulose membranes, and probed with a GFP antibody. The positions of molecular mass markers are shown on the *left* of the first immunoblot, whereas the *numbers* on the *right* relate to the number of mPEG-modified cysteines. The *graphs* show means ± S.E. of normalized *S*-acylation of WT and mutant SNAP25 proteins with HA zDHHC7 or HA zDHHC17; *filled circles* represent individual samples (*n* = 6 or 9 different cell samples for each condition). Statistical analysis (ANOVA followed by a Tukey's *post hoc* test) was performed to reveal significant differences within subgroups of conditions (*WT versus C85L* or *C92L* indicated directly above the *bars*, or *C85L versus C92L* indicated on the *graphs* above a *plain line*). *, *p* < 0.05; **, *p* < 0.01; ***, *p* < 0.001. *WB*, Western blot.

We then studied the *S*-acylation profile of EGFP-SNAP25 WT and mini-linker deletion mutants when co-expressed with zDHHC7 or zDHHC17 using the newly characterized mPEG-Click technique. The mPEG-Click results were consistent with the results obtained with the alkyne IR dye click technique, and the deletions had little effect on *S*-acylation catalyzed by zDHHC7 (Fig. 3*A*). In contrast, for *S*-acylation mediated by zDHHC17, the progressive shortening of the linker region induced an equally progressive disappearance of the PEGylated bands, consistent with a reduction in *S*-acylation (Fig. 3*A*). Our hypothesis is that this loss of *S*-acylation occurs because of reduced accessibility of cysteines in SNAP25 to the active site of zDHHC17 (*i.e.* that the binding site and catalytic site of zDHHC17 are separated by a minimal distance, which also requires a minimal separation of the zDABM and cysteine-rich domain of SNAP25). We reasoned that cysteines in SNAP25 that are closer to the zDABM (*e.g.* Cys^92^) should become inaccessible to the zDHHC17 active site before cysteines that are further away from the zDABM (*e.g.* Cys^85^) as the mini-linker is shortened. To test this, we introduced Cys-to-Leu mutations within two of the deletion mutants that showed a partial but not complete loss of *S*-acylation by zDHHC17 (Δ(101–105) and Δ(101–106); Fig. 1*A*). The cysteines in SNAP25 were replaced with leucine residues rather than alanine or serine because our previous work showed that the hydrophobicity of the cysteines is important for initial membrane interaction of SNAP25 prior to *S*-acylation. This hydrophobicity is preserved by leucine substitutions but not alanine or serine (26). Either the first cysteine of the *S*-acylation domain (Cys^85^) or the last one (Cys^92^) was therefore mutated to leucine. Interestingly, when co-expressed with zDHHC17, the C85L mutation had more impact on the *S*-acylation profile of both of the deletion mutants than the C92L mutation (Fig. 3*B*), showing that Cys^85^ is more efficiently *S*-acylated within the deletion mutants than Cys^92^. This was clearly different from WT SNAP25, in which the C92L mutation decreased *S*-acylation by zDHHC17 more than the C85L mutation (Fig. 3*B*). Overall, these results suggest that Cys^92^ becomes more inaccessible for *S*-acylation when the mini-linker sequence is shortened, consistent with it being closer to the zDABM zDHHC17-binding site.

### Efficient S-acylation of SNAP25 is disrupted by introducing structure into the mini-linker sequence

**Figure 4.**
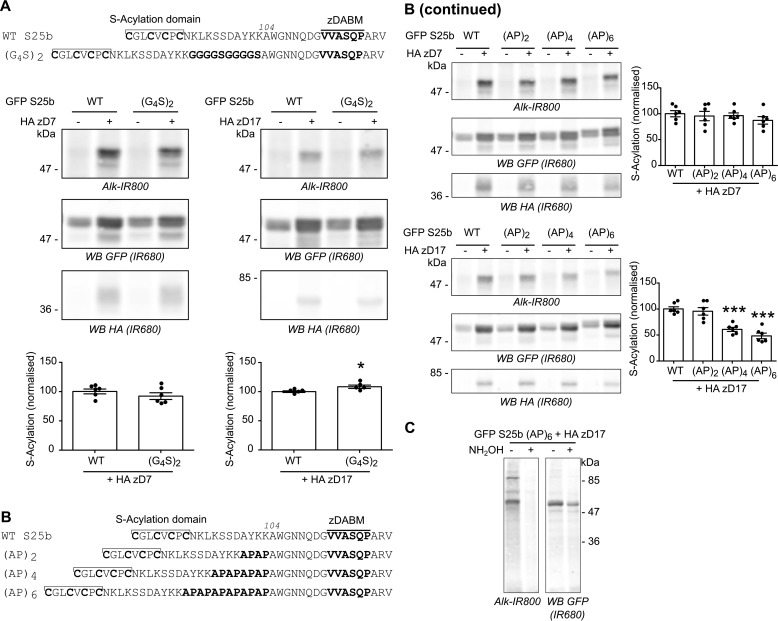
**Differential effects of flexible *versus* rigid sequences inserted into the linker domain of SNAP25.** *A*, *top panel*, schematic showing the introduction of a duplicated flexible sequence composed of four glycines and one serine (G_4_S)_2_ at position 104 of EGFP SNAP25b. *Middle* and *bottom panels*, HEK293T cells were transfected with either EGFP SNAP25b WT (*GFP S25b WT*) or the (G_4_S)_2_ insertion mutant together with a plasmid encoding HA zDHHC7 (+ *HA zD7*), HA zDHHC17 (+ *HA zD17*), or a control pEF-BOS HA plasmid (− *HA zD7*, − *HA zD17*). The cells were then incubated with C16:0 azide, and proteins incorporating the fatty acid azide were labeled by click chemistry using an alkyne-800 IR dye (*Alk-IR800*). Isolated proteins were resolved by SDS-PAGE and transferred to nitrocellulose membranes. Representative images are shown (*middle panel*): click chemistry signal (*top row*, *Alk IR800*), GFP (*middle row*, *IR680*), and HA (*bottom row*, *IR680*) immunoblots. *Graphs* (*bottom panel*) show means ± S.E. of normalized *S*-acylation with HA zDHHC7 or HA zDHHC17; *filled circles* represent individual samples (*n* = 6 different cell samples for each condition). Statistical analysis (Student's *t* test) showed no significant difference between the *S*-acylation of WT *versus* (G_4_S)_2_ by zDHHC7, whereas there was a significant difference of the *S*-acylation of WT *versus* (G_4_S)_2_ by zDHHC17. *, *p* < 0.05. *B*, schematic showing the introduction of (AP)_2_, (AP)_4_, or (AP)_6_ at position 104 of EGFP SNAP25b. *B (continued)*, HEK293T cells were transfected with either EGFP SNAP25b WT (*GFP S25b WT*) or the (AP) insertion mutants ((AP)_2_, (AP)_4_, and (AP)_6_) together with a plasmid encoding HA zDHHC7 (+ *HA zD7*), HA zDHHC17 (+ *HA zD17*), or a control pEF-BOS HA plasmid (− *HA zD7*, − *HA zD17*). The cells were then incubated with C16:0-azide, and proteins incorporating the fatty acid azide were labeled by click chemistry using an alkyne-800 IR dye. Proteins were resolved by SDS-PAGE and transferred to nitrocellulose membranes. Representative images are shown (*left column*): click chemistry signal (*top row*, *IR800*), GFP (*middle row*, *IR680*), and HA (*bottom row*, I*R680*) immunoblots. *Graphs* (*right column*) show means ± S.E. of normalized *S*-acylation with HA zDHHC7 or HA zDHHC17; *filled circles* represent individual samples (*n* = 6 different cell samples for each condition). Statistical analysis (ANOVA) showed no significant difference between the *S*-acylation of WT *versus* any of the (AP) insertion mutants by zDHHC7, whereas there was a significant difference of the *S*-acylation of WT *versus* (AP)_4_ and (AP)_6_ by zDHHC17. ***, *p* < 0.001. *C*, HEK293T cells were transfected with EGFP SNAP25b (AP)_6_ (*GFP S25b (AP)_6_*) together with a plasmid encoding HA zDHHC17 (+ *HA zD17*). The cells were then metabolically labeled with C16:0 azide followed by click chemistry using an alkyne-800 IR dye (*Alk-IR800*). Cell samples were then concentrated by acetone precipitation, aliquoted, and incubated with an equal volume of either 2 m hydroxylamine (+ *NH_2_OH*) or 2 m Tris (− *NH_2_OH*) for 1 h at room temperature. They were then resolved and analyzed by SDS-PAGE. The *left panel* shows the click chemistry signal (*Alk-IR800*), whereas the *right panel* shows the anti-GFP immunoblot (*IR680*). The positions of molecular mass markers (in kDa) are shown on the *right* or *left side* of all immunoblots. *WB*, Western blot.

Because *S*-acylation was disrupted by introducing short deletions to the mini-linker region of SNAP25, we investigated whether inserting additional amino acids was also disruptive. The zDABM and mini-linker region of SNAP25 are disordered (19, 27), and when disorder was maintained through the insertion of a 10-amino acid (G_4_S)_2_ flexible sequence (GGGGSGGGGS) (28), there was no effect on *S*-acylation mediated by zDHHC7 and a very slight but significant increase in *S*-acylation by zDHHC17 (Fig. 4*A*). In contrast, when a more rigid amino acid sequence was introduced at the same position within SNAP25, consisting of Ala-Pro repeats (SNAP25b (AP)_2_, (AP)_4_, and (AP)_6_) (28), there was a clear effect on *S*-acylation mediated by zDHHC17 but not zDHHC7 (Fig. 4*B*). Specifically, the insertion of the 8-amino acid sequence (AP)_4_ or of the 12-amino acid sequence (AP)_6_ significantly decreased SNAP25 *S*-acylation, consistent with a displacement of the *S*-acylation site from the active site of zDHHC17. We also confirmed that the bond linking the C16:0-azide to the (AP)_6_ mutant protein was a thioester because hydroxylamine treatment induced its hydrolysis (Fig. 4*C*). Considering this together with the previous data, we can conclude that the distance between the *S*-acylation domain and the zDABM sequence of SNAP25b is critical for its *S*-acylation by zDHHC17 and that whereas additional flexible amino acid sequences are tolerated, rigid sequences that introduce structural constraints are inhibitory.

### Replacing the mini-linker region with a flexible linker does not affect SNAP25 S-acylation by zDHHC17

**Figure 5.**
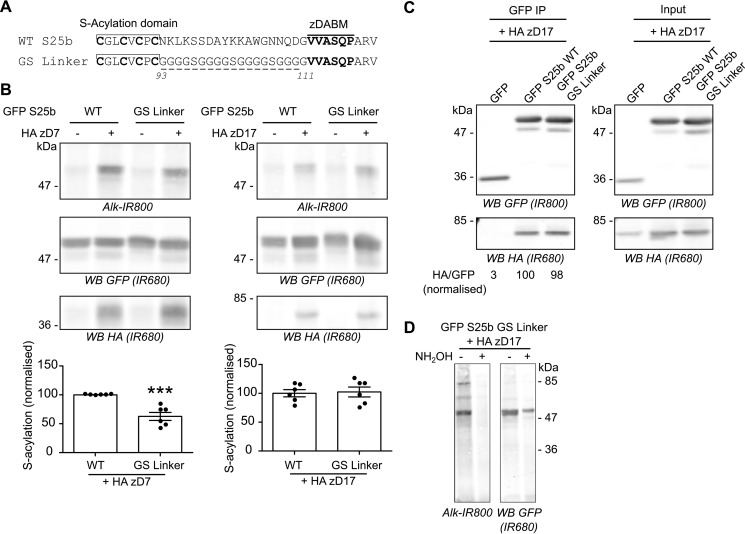
**Replacing the linker region of SNAP25 with a flexible glycine-serine linker of the same length has no effect on SNAP25 *S*-acylation by zDHHC17.** *A*, amino acids (*underlined*) between the *S*-acylation domain and the zDABM of EGFP SNAP25b (*WT S25b*) were replaced by several copies of the flexible GGGGS sequence to create the GS linker mutant. *B*, HEK293T cells were transfected with either EGFP SNAP25b WT (*GFP S25b WT*) or the GS linker mutant together with a plasmid encoding HA zDHHC7 (+ *HA zD7*), HA zDHHC17 (+ *HA zD17*), or a control pEF-BOS HA plasmid (− *HA zD7*, − *HA zD17*). The cells were then incubated with C16:0-azide, and proteins incorporating the fatty acid azide were labeled by click chemistry using an alkyne-800 IR dye. Isolated proteins were resolved by SDS-PAGE and transferred to nitrocellulose membranes. Representative images are shown: click chemistry signal (*top row*, *Alk IR800*), GFP (*middle row*, *IR680*), and HA (*bottom row*, *IR680*) immunoblots. *Graphs* (*bottom panel*) show means ± S.E. of normalized *S*-acylation with HA zDHHC7 (*HA zD7*) or HA zDHHC17 (*HA zD17*); *filled circles* represent individual samples (*n* = 6 different cell samples for each condition). Statistical analysis (Student's *t* test) showed a significant difference between the *S*-acylation of WT *versus* the GS linker mutant by zDHHC7, whereas there was no significant difference of the *S*-acylation of WT *versus* the GS linker mutant by zDHHC17. ***, *p* < 0.001. *C*, HEK293T cells were transfected with EGFP SNAP25b WT (*GFP S25b WT*), GS linker mutant (*GFP S25 GS Linker*), or a GFP control plasmid together with HA zDHHC17 (+ *HA zD17*). Lysates (*right panel*, Input) were immunoprecipitated (*IP*) with a GFP antibody (*left panel*), separated by SDS-PAGE, and transferred to nitrocellulose membranes. The membranes were probed with GFP (*top panel*, *IR800*) and HA (*bottom panel*, *IR680*) antibodies. The ratio between the HA signal and the GFP signal was quantified for the immunoprecipitated samples and is indicated at the *bottom. D*, HEK293T cells were co-transfected with a plasmid encoding EGFP SNAP25b GS linker (*GFP S25b GS Linker*) together with a plasmid encoding HA zDHHC17 (+ *HA zD17*). The cells were metabolically labeled with C16:0 azide followed by click chemistry using an alkyne-800 IR dye (*Alk-IR800*). The cell samples were then concentrated by acetone precipitation, aliquoted, and incubated with an equal volume of either 2 m hydroxylamine (+ *NH_2_OH*) or 2 m Tris (− *NH_2_OH*) for 1 h at room temperature. They were then resolved and analyzed by SDS-PAGE. The *left panel* shows the click chemistry signal (*Alk-IR800*), whereas the *right panel* shows the anti-GFP immunoblot (*IR680*). The positions of molecular mass markers (in kDa) are shown on the *right* or *left* side of all immunoblots. *WB*, Western blot.

The previous analyses have shown that the length of the mini-linker sequence is an important factor for efficient *S*-acylation of SNAP25 by zDHHC17. To examine whether the sequence of the mini-linker is also important, this region (positions 93–111) was entirely replaced with a glycine-serine (GS) linker of the same length (Fig. 5*A*). Unexpectedly, this mutant showed a partial loss of *S*-acylation mediated by zDHHC7, which might reflect a difference in membrane affinity (see “Discussion”). However, this replacement linker sequence had no effect on SNAP25 *S*-acylation by zDHHC17 (Fig. 5*B*). Accordingly, we found that the GS mutant could co-IP zDHHC17 as efficiently as WT SNAP25, showing that the interaction between SNAP25 and zDHHC17 is not dependent on the exact sequence of the mini-linker region (Fig. 5*C*). Fig. 5*D* confirms that C16:0-azide groups are linked to cysteines via thioester bonds on the GS linker mutant because the click signal disappeared after treatment of the samples with hydroxylamine.

**Figure 6.**
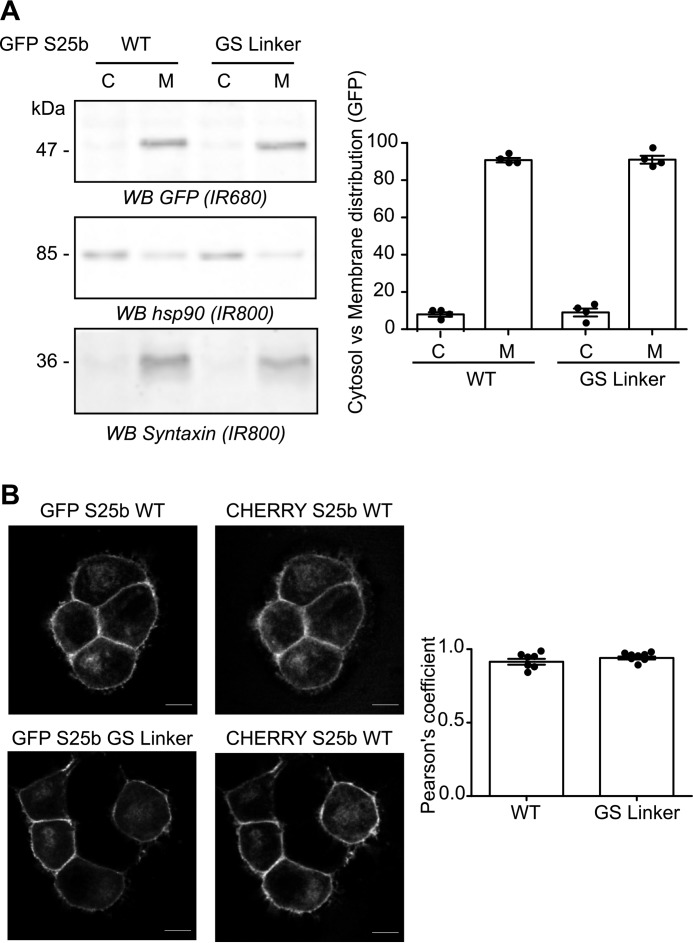
**Replacing the linker region of SNAP25 with a flexible linker does not affect its localization in PC12 cells.** *A*, PC12 cells were transfected with either EGFP SNAP25b WT or the GS linker mutant (*GS Linker*). The cells were fractioned into cytosol (*C*) and membrane (*M*) fractions. The samples were separated by SDS-PAGE and transferred to nitrocellulose membranes. Representative images are shown (*left column*): GFP (*top row*, *IR680*), Hsp90 (*middle row*, *IR800*), and Syntaxin 1a (*bottom row*, *IR800*). The positions of molecular mass markers (in kDa) are shown on the *left side* of all immunoblots. The *graph* (*right panel*) shows means ± S.E. of the cytosol *versus* membrane distribution of the EGFP-tagged proteins; *filled circles* represent individual samples (*n* = 4 fractionations for each condition). Statistical analysis (Student's *t* test) showed no significant difference in the distribution of both constructs. *B*, PC12 cells were transfected with either EGFP SNAP25b WT (*GFP S25b WT*) or the GS linker mutant (*GFP S25b GS Linker*) together with a mCHERRY SNAP25b WT plasmid (*CHERRY S25 WT*). The *middle panel* shows the localization of the WT mCHERRY-tagged SNAP25b, and the *left panel* shows the expression of the co-expressed WT or mutant EGFP-tagged SNAP25b proteins. *Scale bars*, 5 μm. The *graph* (*right panel*) represents means ± S.E. of the Pearson's co-localization coefficient of the GFP signal *versus* mCHERRY signal for both conditions; *filled circles* represent individual images (*n* = 7 cells for each condition). Statistical analysis (Student's *t* test) showed no significant difference between the two constructs. *WB*, Western blot.

Although the replacement linker had no effect on *S*-acylation by zDHHC17, it is possible that it could impact the localization of SNAP25. To examine the effect on localization, we performed experiments in PC12 cells. EGFP-SNAP25 is efficiently *S*-acylated by endogenous zDHHC enzymes in this cell line, and mutation of the zDABM blocks *S*-acylation, demonstrating dependence on endogenous zDHHC17 (23, 29). In addition, SNAP25 is endogenously expressed in PC12 cells, and overexpressed EGFP-SNAP25 localizes correctly to the plasma membrane and endosome/*trans*-Golgi network compartment in these cells (30). Cellular fractionation showed that the GS linker mutant associated with membranes to the same extent as WT SNAP25 in PC12 cells (Fig. 6*A*). Moreover, confocal imaging and analysis also revealed that the mini-linker mutations had no effect on the intracellular localization: both EGFP-tagged SNAP25 WT and GS linker mutant co-localized to the same extent with a mcherry-tagged SNAP25 protein when co-expressed in PC12 cells (Fig. 6*B*). Altogether, these results show that the exact amino acid sequence of the mini-linker region of SNAP25 is not important for its *S*-acylation by zDHHC17 or subsequent targeting to the plasma membrane and endosomal compartments.

### Increasing the affinity of the zDABM for zDHHC17 leads to reduced S-acylation efficiency

**Figure 7.**
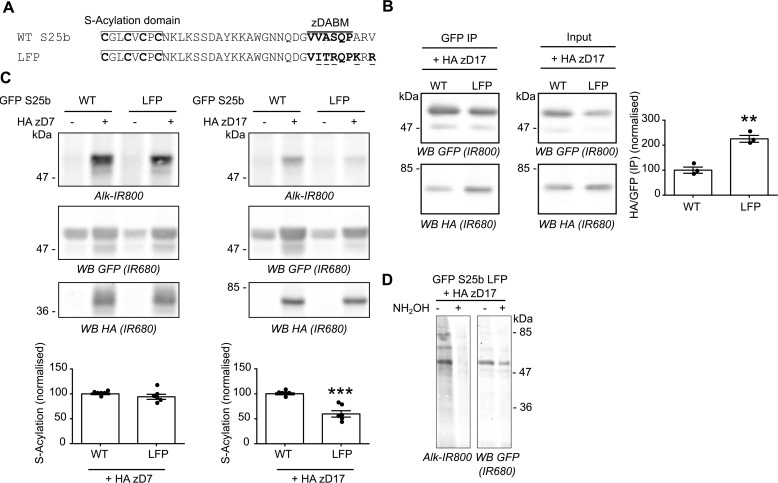
**Increasing the affinity of SNAP25b for zDHHC17 results in decreased *S*-acylation.** *A*, amino acids within the zDABM of SNAP25 were mutated (*underlined*) according to the LFP sequence to increase its affinity for zDHHC17. *B*, HEK293T cells were transfected with either EGFP SNAP25b WT or the LFP mutant together with HA zDHHC17 (+ *HA zD17*). Lysates (*Input*) were immunoprecipitated (*IP*) with a GFP antibody, separated by SDS-PAGE, and transferred to nitrocellulose membranes. Representative images are shown in the *left panel.* Membranes were probed with GFP (*top row*, *IR800*) or HA (*bottom row*, *IR680*) antibodies. The ratio between the HA signal and the GFP signal was quantified for the immunoprecipitated samples. The *graph* (*right panel*) shows means ± S.E. of this ratio; *filled circles* represent individual samples (*n* = 3 immunoprecipitations from different cells for each condition). Statistical analysis (Student's *t* test) showed a significant difference between the interaction of zDHHC17 with SNAP25 WT *versus* the LFP mutant. **, *p* < 0.01. *C*, HEK293T cells were transfected with either EGFP SNAP25b WT (*GFP S25b WT*) or the LFP mutant together with a plasmid encoding HA zDHHC7 (+ *HA zD7*), HA zDHHC17 (+ *HA zD17*), or a control pEF-BOS HA plasmid (− *HA zD7*, − *HA zD17*). The cells were then incubated with C16:0-azide, and proteins incorporating the fatty acid azide were labeled by click chemistry using an alkyne-800 IR dye. Isolated proteins were resolved by SDS-PAGE and transferred to nitrocellulose membranes. Representative images are shown (*top panel*): click chemistry signal (*top row*, *Alk IR800*), GFP (*middle row*, *IR680*), and HA (*bottom row*, *IR680*) immunoblots. *Graphs* (*bottom panel*) show means ± S.E. of normalized *S*-acylation with HA zDHHC7 (+ *HA zD7*) or HA zDHHC17 (+ *HA zD17*); *filled circles* represent individual samples (*n* = 6 different cell samples for each condition). Statistical analysis (Student's *t* test) showed no significant difference between the *S*-acylation of WT *versus* LFP by zDHHC7, whereas there was a significant difference of the *S*-acylation of WT *versus* LFP by zDHHC17. ***, *p* < 0.001. *D*, HEK293T cells were co-transfected with a plasmid encoding EGFP SNAP25b LFP (*GFP S25b LFP*) together with a plasmid encoding HA zDHHC17 (+ *HA zD17*). The cells were metabolically labeled with C16:0 azide followed by click chemistry using an alkyne-800 IR dye (*Alk-IR800*). Cell samples were then concentrated by acetone precipitation, aliquoted, and incubated with an equal volume of either 2 m hydroxylamine (+ *NH_2_OH*) or 2 m Tris (− *NH_2_OH*) for 1 h at room temperature. They were then resolved and analyzed by SDS-PAGE. The *left panel* shows the click chemistry signal (*Alk-IR800*), whereas the *right panel* shows the anti-GFP immunoblot (*IR680*). The positions of molecular mass markers (in kDa) are shown on the *right* or *left side* of all immunoblots. *WB*, Western blot.

Lemonidis *et al.* (21) have previously defined the potentially optimal zDABM amino acid sequence for the binding of substrates to the zDHHC17 ANK domain. A “favorable peptide” sequence was synthesized based on the sequence rules defined for the SNAP25 zDABM and found to interact more strongly than a WT SNAP25 zDABM peptide with the soluble ANK domain of zDHHC17 (21). We therefore modified the zDABM of SNAP25 to include most of the amino acids of the favorable peptide (mutant referred to herein as the Lemonidis favorable peptide, or LFP) (Fig. 7*A*). We found that these amino acid substitutions indeed led to a marked increase in co-IP of HA-zDHHC17 (Fig. 7*B*), confirming that the strength of interaction was increased. When *S*-acylation of this high-affinity mutant was examined, it was found that there was no effect on *S*-acylation mediated by zDHHC7, whereas the LFP substitutions significantly decreased the *S*-acylation of SNAP25 by zDHHC17 (Fig. 7*C*), showing that a higher-affinity interaction between the enzyme and substrate was actually detrimental for *S*-acylation of SNAP25 in cells. Hydroxylamine treatment of the samples induced a disappearance of the click signal (Fig. 7*D*), showing that C16:0-azide is bound to the LFP mutant through thioester bonds, as expected.

### The insertion and deletion mutants Δ(101–108) and (AP)_6_ show a reduced membrane association when expressed in PC12 cells

**Figure 8.**
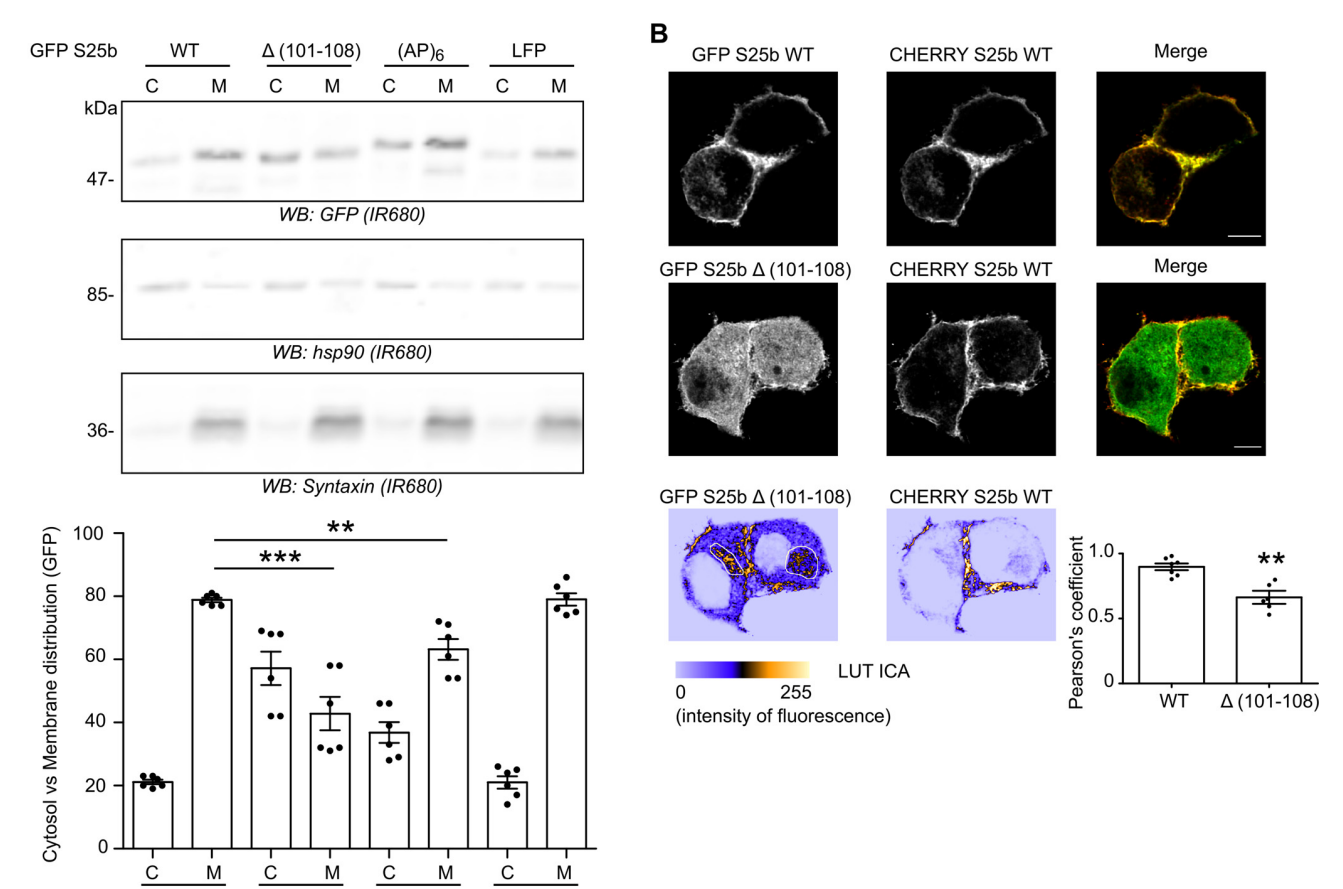
**Modifying the length of the mini-linker affects SNAP25 membrane association in PC12 cells, whereas increasing its affinity for zDHHC17 does not.** *A*, PC12 cells were transfected with either EGFP SNAP25b WT or the following mutants: Δ(101–108), (AP)_6_, or LFP. The cells were fractioned into cytosol (*C*) and membrane (*M*) fractions. The samples were separated by SDS-PAGE and transferred to nitrocellulose membranes. Representative images are shown (*top panel*): GFP (*top row*, *IR680*), Hsp90 (*middle row*, *IR680*), and Syntaxin (*bottom row*, *IR680*). The positions of molecular mass markers are shown on the *left side* of all immunoblots. The *graph* (*bottom panel*) shows means ± S.E. of the cytosol *versus* membrane distribution of the EGFP-tagged proteins; *filled circles* represent individual samples (*n* = 6 different fractionations for each condition). Statistical analysis (ANOVA) showed a significant difference for the membrane association of GFP SNAP25 WT *versus* Δ(101–108) and (AP)_6_. ***, *p* < 0.001; *, *p* < 0.01. *B*, PC12 cells were transfected with either EGFP SNAP25b WT (*GFP S25b WT*) or the Δ(101–108) mutant (*GFP S25b* Δ*(101–108)*) together with a mCHERRY SNAP25b WT plasmid (*CHERRY S25b WT*). The *middle panels* show the localization of the WT mCHERRY-tagged SNAP25b, and the *left panels* show the expression of the co-expressed WT or mutant EGFP-tagged SNAP25b proteins. The *right panels* represent the merged image with GFP SNAP25b WT and mutant proteins in *green* and CHERRY SNAP25b WT protein in *red. Scale bars*, 5 μm. The *graph* (*bottom right panel*) represents means ± S.E. of the Pearson's coefficient of the GFP signal *versus* mCHERRY signal for both constructs; *filled circles* represent individual images (*n* = 5–7 cells for each condition). Statistical analysis (Student's *t* test) showed a significant difference between the localization of the WT protein *versus* the Δ(101–108) deletion mutant. **, *p* < 0.01. The *two bottom confocal images* are displayed with the LUT ICA, which is proportional to the intensity of fluorescence. These images were taken from the same cells as above (cells co-expressing GFP S25b Δ(101–108) and CHERRY S25b WT) but at a different depth within the cells to visualize the Golgi region (manually drawn on the GFP S25b Δ(101–108) image). This pseudo-coloring highlights the accumulation of the Δ(101–108) deletion mutant in the Golgi region of the cells. *WB*, Western blot.

Our previous work has shown that EGFP-SNAP25 is efficiently *S*-acylated in PC12 cells, most likely by endogenous zDHHC17. Thus, we examined the membrane targeting of SNAP25 WT or the Δ(101–108), (AP)_6_, or LFP mutants in these cells in the absence of zDHHC enzyme co-expression. Fig. 8*A* shows that both the Δ(101–108) and (AP)_6_ mutants had a significantly increased presence in purified cytosolic fractions and a decreased association with membrane fractions compared with WT SNAP25 when expressed in PC12 cells. These results are consistent with the reduced *S*-acylation observed for these mutants when co-expressed with zDHHC17 in HEK293T cells. In contrast, the LFP mutant associated with membranes to the same extent as the WT protein when expressed in PC12 cells. This result suggests that although a higher affinity for zDHHC17 interferes with the *S*-acylation process in HEK293T cells, this does not have a major impact on steady-state SNAP25 membrane association in PC12 cells. The cytosol and membrane fractions were also probed with antibodies against Hsp90 and syntaxin, a soluble and transmembrane protein, respectively. Finally, we examined the intracellular localization of the Δ(101–108) mutant, which is the most affected in its membrane association, by confocal microscopy (Fig. 8*B*). This confirmed the increased cytosolic localization of this mutant compared with WT. Interestingly, we also detected an accumulation of this mutant in the Golgi region of the cells (*lower panel*), consistent with the idea that this mutant retains an underlying membrane affinity and can interact with zDHHC17 (which is Golgi-localized) but has a defect in subsequent *S*-acylation.

## Discussion

Identification of the substrate networks of individual zDHHC enzymes is a major requirement for progress of the *S*-acylation field. Experiments to determine these substrate networks (*e.g.* by zDHHC depletion and quantitative proteomics) will be complicated by the observation that zDHHC enzymes can have overlapping substrate selectivity (1). SNAP25, the focus of the current study, interacts with and is *S*-acylated by zDHHC17 (6, 17, 19, 21, 23, 29). However, co-expression experiments have revealed that this protein can also be modified by zDHHC2, zDHHC3, zDHHC7, and zDHHC15 (6, 22). Indeed, zDHHC3 and zDHHC7 *S*-acylate SNAP25 to a greater extent than zDHHC17, despite zDHHC3/7 displaying no detectable interaction with SNAP25 (17). Furthermore, zDHHC3/7 can modify a diverse set of substrates that have no obvious sequence or structural similarities. Based on this, we believe that zDHHC3/7 are high-activity/low-selectivity enzymes that modify accessible and reactive cysteines on proteins that co-localize at the Golgi (*e.g.* during trafficking of newly synthesized proteins), in the absence of any specific enzyme-substrate recognition. In contrast, zDHHC17 is a low-activity/high-selectivity enzyme that depends upon specific interactions with its substrate proteins to mediate *S*-acylation. This idea is supported by several observations, including (i) removal of the substrate-binding ANK domain prevents *S*-acylation by zDHHC17 (17) and (ii) mutation of the zDABM motif of SNAP25 prevents *S*-acylation by zDHHC17 (19, 21–23). Indeed, it is interesting to note that although zDHHC3 and zDHHC7 robustly *S*-acylate SNAP25 in co-expression experiments, mutation of the zDABM in SNAP25 leads to a loss of membrane binding (and thus presumably *S*-acylation) in PC12 cells (22), demonstrating the importance of zDHHC17 for SNAP25 *S*-acylation in this cell type at least.

The current study sought to determine features of SNAP25 that are important for the coupling of binding to zDHHC17 with subsequent *S*-acylation. This question is important because we showed that zDHHC17 interacts with zDABM sequences from a diverse set of proteins and that several of these binders are not known to be *S*-acylated (21). Thus, we were interested in the features of SNAP25 that allow it to be a zDHHC17 *S*-acylation substrate. The results of the study showed that there is a minimum separation length required between the cysteine-rich domain and zDABM for *S*-acylation by zDHHC17. These two regions of SNAP25 are separated by 19 amino acids (residues 93–111); because this part of SNAP25 is thought to be disordered (27), an extended peptide sequence of this length would be in the region of 7.6 nm (assuming 0.4 nm/amino acid (31)). Removal of four amino acids was tolerated, whereas removal of five or more amino acids from this linker region reduced *S*-acylation, suggesting a minimal required separation in the order of 6 nm between zDABM and cysteines for efficient *S*-acylation. Although the structure of full-length zDHHC17 has not been reported, these approximate calculations of the displacement of the ANK domain and DHHC domain of zDHHC17 can be tested when structural information is available. By using mPEG-Click analysis of C85L and C92L mutants of Δ(101–105) and Δ(101–106), it was found that the C85L mutation had a greater effect than the C92L mutation in the context of the deletion mutants, whereas there was no obvious difference between these mutations in full-length SNAP25. This supports that idea that as the mini-linker is shortened, cysteines closer to the zDABM (*i.e.* Cys^92^) become less accessible for *S*-acylation before cysteines further from the zDABM (*i.e.* Cys^85^), thus explaining why Cys^85^ mutation has a greater effect than Cys^92^ mutation on *S*-acylation of the linker deletion mutants. Additionally, our previous work suggested that the hydrophobic cysteine-rich domain of SNAP25 mediates initial transient association of SNAP25 with Golgi membranes, allowing the protein to interact with its target zDHHC enzyme (zDHHC17) (23). Thus, we predicted that the Δ(101–108) mutant that has a defect in *S*-acylation but not in its interaction with zDHHC17 might show some accumulation at Golgi membranes when expressed in PC12 cells. Confocal analysis indeed showed accumulation of this protein in a Golgi-like region of the cell.

The mPEG-Click technique newly described here was used to generate additional data to the ones obtained with the widely used dye-click technique. Indeed, mPEG-Click reveals the number of *S*-acylated cysteines and also differentiates between mutations affecting the overall *S*-acylation of a protein and mutations affecting the *S*-acylation of specific cysteines within a protein. Coupled with pulse-chase and time-course analyses, mPEG-Click could be used to study the dynamics and progress of cysteine *S*-acylation/deacylation within multiply *S*-acylated proteins. Because the *S*-acylation profile of a protein is simply revealed with antibodies against this protein, overexpression of the protein of interest is not strictly necessary, as long as the antibody is sensitive enough to detect the endogenous protein, and the epitopes are not masked by the addition of the mPEG group to the *S*-acylated cysteines. In our opinion, mPEG-Click might provide an easy and straightforward means to investigate the *S*-acylation status of any endogenous or overexpressed protein in various cell types. Previous work used a similar band shift brought about by ligation of azido-TAMRA-PEG-Biotin to alkynyl cholesterol analogues to study cholesterylation of Sonic Hedgehog (32).

This work describes the first use of mPEG-Click methodology to study the dynamics of protein *S*-acylation. However, another PEG-based assay, variably referred to as PEG-switch (33), acyl-PEG exchange (34), or acyl-PEGyl exchange gel shift (7), is commonly used in the *S*-acylation field. This methodology is different from mPEG-Click because it involves removal of unlabeled acyl chains with hydroxylamine followed by labeling of the thus freed cysteines by a cysteine-reactive reagent such as PEG-maleimide. mPEG-Click and acyl-PEG switch both have distinct advantages; for example, acyl-PEG switch is well-suited to study the extent of *S*-acylation on endogenous proteins in cell and tissue extracts, whereas mPEG-Click has wider scope for the analysis of *S*-acylation dynamics in living cells.

In contrast to mutations that shortened the mini-linker region, addition of a 10-amino acid flexible glycine-serine linker had no major effect on *S*-acylation. This suggests that whereas there is a strict requirement for a minimal separation distance between zDABM and *S*-acylated cysteines, there is more flexibility around the maximal length, presumably because a longer disordered and flexible linker can adopt a spatial orientation that can simultaneously dock the zDABM and cysteines of a substrate into the ANK domain and DHHC domain of zDHHC17, respectively. In contrast, the insertion of rigid inflexible alanine-proline sequences into the mini-linker region of SNAP25 perturbed *S*-acylation by zDHHC17, presumably by limiting spatial flexibility of the linker region and hence the spatial optimization of zDABM and cysteine-rich domain of this protein.

Interestingly, analysis of the GS linker mutant that had a complete replacement of the 19-amino acid linker with a similar length GS linker suggested that the mini-linker sequence is not important for either *S*-acylation or intracellular targeting of SNAP25. It was notable, however, that this GS linker mutant was less efficiently *S*-acylated by zDHHC7. Our previous work suggested that the hydrophobic cysteine-rich domain of SNAP25 (residues 85–92) plays an important role in initial membrane interaction of SNAP25 with Golgi membranes prior to *S*-acylation (23). In contrast, a study by the Lang group (35) suggested a role for positively charged amino acids in the (large) linker domain in mediating this initial membrane interaction. However, it is worthwhile further reflecting on the proposed role of positively charged amino acids in initial membrane interaction of SNAP25 (35). The results of the current study clearly show that lysine residues in the mini-linker are dispensable for *S*-acylation mediated by zDHHC17 and for membrane association and intracellular targeting in PC12 cells. Furthermore, any positively charged residues upstream of the cysteine-rich domain are also dispensable as the minimal *S*-acylation and membrane targeting domain in SNAP25b is amino acids 85–120 (36). Thus, it is likely that any role played by positively charged amino acids of SNAP25 is secondary to the prominent role of the hydrophobic cysteine-rich domain in initial membrane targeting of this protein (23). Indeed, the importance of the nonacylated cysteine-rich domain in membrane interactions of SNAP25 was further shown by a recent study (37). The effect of mini-linker replacement on *S*-acylation by zDHHC7 may reflect the idea that this enzyme does not interact with SNAP25 directly (17) but rather mediates “stochastic” *S*-acylation of this protein when it is present at the membrane. Thus, a slight loss in membrane affinity caused by mini-linker replacement could lead to the reduced *S*-acylation seen with zDHHC7, whereas a slight reduction in membrane affinity may still be sufficient to promote efficient zDHHC17–SNAP25 interaction and subsequent *S*-acylation.

The affinity of the zDABM of SNAP25 for the ANK domain of zDHHC17 was calculated to be ∼11 μm, although full-length SNAP25 had a higher affinity (20). This interaction affinity is clearly sufficient to allow robust isolation of the protein complex by co-IP. However, we were interested in whether enhancing the affinity of interaction would lead to higher levels of *S*-acylation. To test this idea, we used data from a previous study by our group (21) to generate a SNAP25 mutant, which was predicted to have an increased affinity for the ANK domain of zDHHC17. In support of this idea, the mutant captured higher levels of zDHHC17 by co-IP. Despite this increased binding capacity, the LFP high-affinity mutant displayed reduced *S*-acylation by zDHHC17 when expressed in HEK293T cells, suggesting that tight binding between zDHHC enzymes and other proteins is not always conducive to efficient *S*-acylation; perhaps tighter binding limits the catalytic turnover of the enzyme. It was interesting to note that although the LFP mutant displayed reduced *S*-acylation by zDHHC17 (but not zDHHC7) in HEK293T cells, there was no significant loss of membrane binding of this mutant in PC12 cells. We believe that this reflects the fact that membrane binding of the LFP mutant in PC12 cells was measured 48 h after transfection and reflects the steady-state distribution of the protein. In contrast, *S*-acylation assays in HEK293T cells are performed over 4 h and are therefore better suited to detect changes in the overall kinetics of *S*-acylation.

In theory, the reduced *S*-acylation signal of specific SNAP25 linker mutants in click chemistry experiments could reflect either a defect in *S*-acylation or an enhanced rate of deacylation. However, in all experiments, where zDHHC17-mediated *S*-acylation was reduced, we showed that there was no effect on *S*-acylation levels of the corresponding mutants co-expressed with zDHHC7. This clearly implies that the linker mutants have a specific defect in *S*-acylation mediated by zDHHC17 rather than any change in deacylation rate.

This study demonstrates that the length and flexible character of the mini-linker region of SNAP25 are important features for efficient *S*-acylation. In contrast, the sequence of the mini-linker is less important for efficient *S*-acylation as long as it confers structural flexibility. In follow-up work, it would be interesting to examine the interaction between zDHHC17 and SNAP25 using purified proteins. This would allow a more detailed analysis of factors such as *K_m_* and *K*_cat_ and how these are affected by changes to the linker region of SNAP25. These analyses would allow a more refined understanding of the enzymology of zDHHC17 to emerge. Future work will also determine whether the sequence of the mini-linker is important for the function of SNAP25 in fusion pore dynamics and exocytosis (38).

## Experimental procedures

### Antibodies

Mouse GFP antibody (Clontech, clone JL8, used at 1:4,000) was obtained from Takara (Saint-Germain-en-Laye, France). Rat HA antibody (Roche, clone 3F10, used at 1:1,000) was from Sigma. Rabbit HSP90 antibody (C45G5, used at 1:1,000) was from Abcam (Cambridge, UK). Mouse anti-Syntaxin antibody (HPC-1 S0664, used at 1:1,000) was from Sigma. IR dye–conjugated secondary antibodies (used at 1:20,000 dilution) were purchased from Li-COR Biosciences (Cambridge, UK).

### Plasmid DNA

cDNA encoding human zDHHC7 and zDHHC17 and the (AP)_6_ and GS linker SNAP25b mutants were synthesized by Thermo Fisher Scientific. The cDNA encoding zDHHC7 and zDHHC17 were subcloned into pEF-BOS HA (6), whereas the SNAP25b constructs were subcloned in pEGFP-C2 (23). WT rat SNAP25b (in pEGFP-C2), SNAP25 mutants with deletions of the linker region, and mcherry-SNAP25 were previously described (23, 30). All other mutants described in this report were generated by site-directed mutagenesis using oligonucleotide primers synthesized by. The validity of all constructs was confirmed by sequencing (Dundee DNA Sequencing Service).

### Cells

HEK293T cells (CRL-3216, ATCC) were grown in Dulbecco's modified Eagle's medium (Thermo Fisher Scientific) supplemented with 10% fetal bovine serum. PC12 cells (CRL-1721, ATCC) were grown in Advanced RPMI 1640 medium (Thermo Fisher Scientific) containing 10% horse serum, 5% fetal bovine serum, and 1% glutamine. All cells were grown at 37 °C in a humidified atmosphere containing 5% CO_2_.

### Cell transfection

For substrate *S*-acylation and immunoprecipitation assays, HEK293T were plated on poly-d-lysine–coated 24-well plates (Corning BioCoat, VWR, UK) and transfected with 0.33 μg of EGFP-SNAP25 plasmid and 0.66 μg of pEF-BOS HA plasmid (either empty as a control or encoding the zDHHC enzymes). 2 μl of polyethylene imine (1 mg/ml stock) (linear polyethylene imine molecular weight 25,000, #43896, Alpha Aesar) was added to the DNA mix, incubated for 20 min, and then added to the cells, which were analyzed the following day.

For cell fractionation and immunofluorescence analyses, PC12 cells were plated on poly-d-lysine–coated 24-well plates or on 12-mm BD poly-d-lysine–coated coverslips (Thermo Fisher Scientific), respectively. The cells were transfected using Lipofectamine 2000 (Invitrogen) with a ratio of 2 μl of Lipofectamine/μg of DNA. For fractionation experiments, 1 μg of EGFP SNAP25 WT or mutants was transfected, whereas 0.2 μg of EGFP SNAP25b WT or mutants was transfected together with 0.2 μg of mCHERRY SNAP25b WT for immunofluorescence analysis. The cells were incubated with the transfection mixture for ∼48 h before analysis.

### Cell labeling with fatty acids

HEK293T cells in 24-well plates were incubated with 100 μm of either palmitic acid (P0500, Sigma) or C16:0-azide (16) in 350 μl of serum-free Dulbecco's modified Eagle's medium supplemented with 1 mg/ml defatted BSA (A7030, Sigma) for 4 h at 37 °C.

### Detection of palmitate labeled probes in S-acylated proteins

The cells were washed once with PBS and then lysed on ice in 100 μl of 50 mm Tris, pH 8.0, containing 0.5% SDS and protease inhibitors (P8340, Sigma). Conjugation of IR800CW Alkyne dye or mPEG5K Alkyne to C16:0-azide was carried out for 1 h at room temperature with end-over-end rotation by adding an equal volume (100 μl) of freshly prepared click-chemistry reaction mixture containing the following: 5 μm IR dye 800CW Alkyne (929-60002, Li-COR) or 200 μm mPEG5k-Alkyne (JKA3177, Sigma), 4 mm CuSO_4_ (451657, Sigma), 400 μm Tris[(1-benzyl-1*H*-1,2,3-triazol-4-yl)methyl]amine (678937, Sigma), and 8 mm ascorbic acid (A15613, Alpha Aesar) in distilled H_2_O. 67 μl of 4× SDS sample buffer containing 100 mm DTT was then added to the 200-μl sample. Protein samples were incubated at 95 °C for 5 min, and 19 μl was resolved by SDS-PAGE and transferred to nitrocellulose for immunoblotting analysis. Immunoblots were quantified with the ImageStudio software from Li-Cor. Graphs were created and statistical analysis was performed using GraphPad software.

### Quantification and determination of the efficiency of S-acylation

*S*-Acylation was calculated as the ratio between Click signal (IR800) and GFP signal (IR680). This ratio was then normalized to control conditions on the same membrane. For Alkyne mPEG-treated samples, the efficiency of *S*-acylation was calculated as the ratio between the sum of GFP signals corresponding to one, two, three, and four modified cysteines and the sum of all the signals (*i.e.* sum of zero, one, two, three, and four modified cysteines). The value for cells incubated with unlabeled palmitate was then subtracted from the values of similarly transfected cells incubated with C16:0-azide. The efficiency of *S*-acylation was then normalized to control conditions.

### Chemical deacylation

HEK293T cells were transfected and labeled by click chemistry (IR dye 800 CW) as described above. Proteins were precipitated by the addition of 3 volumes of ice-cold acetone and incubation at −20 °C for 30 min. Pellets resulting from a 10-min centrifugation at 16,000 × *g* (4 °C) were then washed three times with ice-cold 70% acetone, air-dried for 10 min, and resuspended in 100 mm HEPES, pH 7.5, 1 mm EDTA, 1% SDS. 20-μl aliquots were then incubated with an equal volume of 2 m Tris, pH 7.5, or 2 m hydroxylamine (pH 7.5) for 1 h at room temperature. The samples were resolved and analyzed by SDS-PAGE.

### Immunoprecipitation

The cells were co-transfected with plasmids encoding EGFP-SNAP25 constructs together with a HA-tagged zDHHC17 construct on 24-well plates. Three identical wells were gathered for each immunoprecipitation. The cells were washed, lysed in 200 μl of PBS, 0.5% Triton X-100 (T8787, Sigma) supplemented with protease inhibitors for 30 min on ice, and clarified by centrifugation at 16,000 × *g* for 10 min at 4 °C. The supernatants were then adjusted to 0.2% Triton X-100 by the addition of 300 μl of cold PBS. Whereas 50 μl of lysate was kept as the input fraction, 450 μl of lysate was added to 8 μl (slurry volume) of washed GFP–Trap–agarose beads (GTA-20, Chromotek). The tubes were incubated on an end-over-end rotator at 4 °C for 1 h. The beads were then washed twice with PBS and eluted with 50 μl of 2× SDS-PAGE sample buffer containing 50 mm DTT with a 5-min incubation at 95 °C.

### Fractionation of PC12 cells

Transfected PC12 cells were detached from the wells in 500 μl of PBS and transferred to Eppendorf tubes. Cell fractionation was performed as described by Baghirova *et al.* (39). Briefly, the cells were washed in PBS; resuspended in 150 μl of buffer A (150 mm NaCl, 50 mm HEPES, pH 7.4, 1 m hexylene glycol (112100, Sigma), 25 μg/ml digitonin (D141, Sigma)) supplemented with protease inhibitors; and rotated end-over-end at 4 °C for 10 min. The supernatant of a 2,000 × *g*, 10-min centrifugation at 4 °C was collected as the cytosolic fraction. The pellet was washed briefly in buffer A before being resuspended by vortexing in 150 μl of buffer B (150 mm NaCl, 50 mm HEPES, pH 7.4, 1 m hexylene glycol, 1% (v/v) Igepal (CA-630, Sigma) supplemented with protease inhibitors. After a 30-min incubation on ice, the lysates were centrifuged at 7,000 × *g* for 10 min at 4 °C; the supernatant was collected as the membrane fraction. Both the cytosolic and membrane fractions were supplemented with 4× SDS-PAGE loading buffer containing 100 mm DTT and incubated at 95 °C for 5 min. The proteins were resolved by loading an equal volume of each fraction by SDS-PAGE and analysis by immunoblotting. The effectiveness of the membrane/cytosol fractionation was assessed by probing with antibodies against Syntaxin and Hsp90.

### Immunofluorescence, confocal microscopy, and image analysis

Transfected cells were washed once with PBS and fixed in 4% formaldehyde for 30 min. The cells were then washed again in PBS and mounted on glass slides in Mowiol. All microscopy analysis was performed on a Leica SP8 confocal microscope; image stacks were acquired with the Lightning function. A single slice was chosen as a representative image. Image quantification was performed with the fiji software. The co-localization threshold tool was used to calculate the Pearson's co-localization coefficient *r*. Statistical analysis was performed with the GraphPad software.

## Author contributions

C. S., J. G., N. C. O. T., and L. H. C. conceptualization; C. S. data curation; C. S. and L. H. C. formal analysis; C. S. and L. H. C. investigation; C. S., N. C. O. T., and L. H. C. methodology; C. S. and L. H. C. writing-original draft; C. S., J. G., N. C. O. T., and L. H. C. writing-review and editing; J. G. and N. C. O. T. resources; N. C. O. T. and L. H. C. funding acquisition; L. H. C. project administration.

## Footnotes

3 The abbreviations used are:

zDHHC: zinc finger DHHC-type palmitoyltransferase
ANK: ankyrin repeat
zDABM: zDHHC ANK binding motif
LFP: Lemonidis favorable peptide
IP: immunoprecipitation
HA: hemagglutinin
ANOVA: analysis of variance.

## References

[1] Chamberlain L. H., and Shipston M. J. 2015) The physiology of protein *S*-acylation. Physiol. Rev. 95, 341–376 10.1152/physrev.00032.2014 25834228 PMC4551212

[2] Salaun C., Greaves J., and Chamberlain L. H. 2010) The intracellular dynamic of protein palmitoylation. J. Cell Biol. 191, 1229–1238 10.1083/jcb.201008160 21187327 PMC3010063

[3] Blanc M., David F. P. A., and van der Goot F. G. 2019) SwissPalm 2: protein *S*-palmitoylation database. Methods Mol. Biol. 2009, 203–214 10.1007/978-1-4939-9532-5_16 31152406

[4] Blanc M., David F., Abrami L., Migliozzi D., Armand F., Bürgi J., and van der Goot F. G. 2015) SwissPalm: protein palmitoylation database. F1000Research 4, 261 10.12688/f1000research.6464.1 26339475 PMC4544385

[5] Sanders S. S., Martin D. D., Butland S. L., Lavallée-Adam M., Calzolari D., Kay C., Yates J. R. 3rd, and Hayden M. R. 2015) Curation of the mammalian palmitoylome indicates a pivotal role for palmitoylation in diseases and disorders of the nervous system and cancers. PLoS Comput. Biol. 11, e1004405 10.1371/journal.pcbi.1004405 26275289 PMC4537140

[6] Fukata M., Fukata Y., Adesnik H., Nicoll R. A., and Bredt D. S. 2004) Identification of PSD-95 palmitoylating enzymes. Neuron 44, 987–996 10.1016/j.neuron.2004.12.005 15603741

[7] Yokoi N., Fukata Y., Sekiya A., Murakami T., Kobayashi K., and Fukata M. 2016) Identification of PSD-95 depalmitoylating enzymes. J. Neurosci. 36, 6431–6444 10.1523/JNEUROSCI.0419-16.2016 27307232 PMC5015780

[8] Noritake J., Fukata Y., Iwanaga T., Hosomi N., Tsutsumi R., Matsuda N., Tani H., Iwanari H., Mochizuki Y., Kodama T., Matsuura Y., Bredt D. S., Hamakubo T., and Fukata M. 2009) Mobile DHHC palmitoylating enzyme mediates activity-sensitive synaptic targeting of PSD-95. J. Cell Biol. 186, 147–160 10.1083/jcb.200903101 19596852 PMC2712995

[9] Thomas G. M., Hayashi T., Chiu S. L., Chen C. M., and Huganir R. L. 2012) Palmitoylation by DHHC5/8 targets GRIP1 to dendritic endosomes to regulate AMPA-R trafficking. Neuron 73, 482–496 10.1016/j.neuron.2011.11.021 22325201 PMC3345505

[10] Ohno Y., Kihara A., Sano T., and Igarashi Y. 2006) Intracellular localization and tissue-specific distribution of human and yeast DHHC cysteine-rich domain-containing proteins. Biochim. Biophys. Acta 1761, 474–483 10.1016/j.bbalip.2006.03.010 16647879

[11] Jennings B. C., and Linder M. E. 2012) DHHC protein *S*-acyltransferases use similar ping-pong kinetic mechanisms but display different acyl-CoA specificities. J. Biol. Chem. 287, 7236–7245 10.1074/jbc.M111.337246 22247542 PMC3293542

[12] Mitchell D. A., Mitchell G., Ling Y., Budde C., and Deschenes R. J. 2010) Mutational analysis of Saccharomyces cerevisiae Erf2 reveals a two-step reaction mechanism for protein palmitoylation by DHHC enzymes. J. Biol. Chem. 285, 38104–38114 10.1074/jbc.M110.169102 20851885 PMC2992244

[13] Rana M. S., Lee C. J., and Banerjee A. 2019) The molecular mechanism of DHHC protein acyltransferases. Biochem. Soc. Trans. 47, 157–167 10.1042/BST20180429 30559274

[14] Rana M. S., Kumar P., Lee C. J., Verardi R., Rajashankar K. R., and Banerjee A. 2018) Fatty acyl recognition and transfer by an integral membrane *S*-acyltransferase. Science 359, eaao6326 10.1126/science.aao6326 29326245 PMC6317078

[15] Liang X., Nazarian A., Erdjument-Bromage H., Bornmann W., Tempst P., and Resh M. D. 2001) Heterogeneous fatty acylation of Src family kinases with polyunsaturated fatty acids regulates raft localization and signal transduction. J. Biol. Chem. 276, 30987–30994 10.1074/jbc.M104018200 11423543

[16] Greaves J., Munro K. R., Davidson S. C., Riviere M., Wojno J., Smith T. K., Tomkinson N. C., and Chamberlain L. H. 2017) Molecular basis of fatty acid selectivity in the zDHHC family of *S*-acyltransferases revealed by click chemistry. Proc. Natl. Acad. Sci. U.S.A. 114, E1365–E1374 10.1073/pnas.1612254114 28167757 PMC5338407

[17] Lemonidis K., Gorleku O. A., Sanchez-Perez M. C., Grefen C., and Chamberlain L. H. 2014) The Golgi *S*-acylation machinery comprises zDHHC enzymes with major differences in substrate affinity and *S*-acylation activity. Mol. Biol. Cell 25, 3870–3883 10.1091/mbc.e14-06-1169 25253725 PMC4244197

[18] Huang K., Sanders S., Singaraja R., Orban P., Cijsouw T., Arstikaitis P., Yanai A., Hayden M. R., and El-Husseini A. 2009) Neuronal palmitoyl acyl transferases exhibit distinct substrate specificity. FASEB J. 23, 2605–2615 10.1096/fj.08-127399 19299482 PMC2717768

[19] Lemonidis K., Sanchez-Perez M. C., and Chamberlain L. H. 2015) Identification of a novel sequence motif recognized by the ankyrin repeat domain of zDHHC17/13 *S*-acyltransferases. J. Biol. Chem. 290, 21939–21950 10.1074/jbc.M115.657668 26198635 PMC4571948

[20] Verardi R., Kim J. S., Ghirlando R., and Banerjee A. 2017) Structural basis for substrate recognition by the ankyrin repeat domain of human DHHC17 palmitoyltransferase. Structure 25, 1337–1347.e6 10.1016/j.str.2017.06.018 28757145 PMC5599134

[21] Lemonidis K., MacLeod R., Baillie G. S., and Chamberlain L. H. 2017) Peptide array-based screening reveals a large number of proteins interacting with the ankyrin-repeat domain of the zDHHC17 *S*-acyltransferase. J. Biol. Chem. 292, 17190–17202 10.1074/jbc.M117.799650 28882895 PMC5655499

[22] Greaves J., Gorleku O. A., Salaun C., and Chamberlain L. H. 2010) Palmitoylation of the SNAP25 protein family: specificity and regulation by DHHC palmitoyl transferases. J. Biol. Chem. 285, 24629–24638 10.1074/jbc.M110.119289 20519516 PMC2915699

[23] Greaves J., Prescott G. R., Fukata Y., Fukata M., Salaun C., and Chamberlain L. H. 2009) The hydrophobic cysteine-rich domain of SNAP25 couples with downstream residues to mediate membrane interactions and recognition by DHHC palmitoyl transferases. Mol. Biol. Cell 20, 1845–1854 10.1091/mbc.e08-09-0944 19158383 PMC2655257

[24] Mitchell D. A., Vasudevan A., Linder M. E., and Deschenes R. J. 2006) Protein palmitoylation by a family of DHHC protein *S*-acyltransferases. J. Lipid Res. 47, 1118–1127 10.1194/jlr.R600007-JLR200 16582420

[25] Fasshauer D., Eliason W. K., Brünger A. T., and Jahn R. 1998) Identification of a minimal core of the synaptic SNARE complex sufficient for reversible assembly and disassembly. Biochemistry 37, 10354–10362 10.1021/bi980542h 9671503

[26] Greaves J., and Chamberlain L. H. 2006) Dual role of the cysteine-string domain in membrane binding and palmitoylation-dependent sorting of the molecular chaperone cysteine-string protein. Mol. Biol. Cell 17, 4748–4759 10.1091/mbc.e06-03-0183 16943324 PMC1635403

[27] Margittai M., Fasshauer D., Pabst S., Jahn R., and Langen R. 2001) Homo- and heterooligomeric SNARE complexes studied by site-directed spin labeling. J. Biol. Chem. 276, 13169–13177 10.1074/jbc.M010653200 11278719

[28] Chen X., Zaro J. L., and Shen W. C. 2013) Fusion protein linkers: property, design and functionality. Adv. Drug Deliver. Rev. 65, 1357–1369 10.1016/j.addr.2012.09.039 23026637 PMC3726540

[29] Greaves J., and Chamberlain L. H. 2010) *S*-Acylation by the DHHC protein family. Biochem. Soc. Trans. 38, 522–524 10.1042/BST0380522 20298214

[30] Greaves J., and Chamberlain L. H. 2011) Differential palmitoylation regulates intracellular patterning of SNAP25. J. Cell Sci. 124, 1351–1360 10.1242/jcs.079095 21429935 PMC3065388

[31] Ainavarapu S. R., Brujic J., Huang H. H., Wiita A. P., Lu H., Li L., Walther K. A., Carrion-Vazquez M., Li H., and Fernandez J. M. 2007) Contour length and refolding rate of a small protein controlled by engineered disulfide bonds. Biophys. J. 92, 225–233 10.1529/biophysj.106.091561 17028145 PMC1697845

[32] Ciepla P., Konitsiotis A. D., Serwa R. A., Masumoto N., Leong W. P., Dallman M. J., Magee A. I., and Tate E. W. 2014) New chemical probes targeting cholesterylation of Sonic Hedgehog in human cells and zebrafish. Chem. Sci. 5, 4249–4259 10.1039/C4SC01600A 25574372 PMC4285107

[33] Howie J., Reilly L., Fraser N. J., Vlachaki Walker J. M., Wypijewski K. J., Ashford M. L., Calaghan S. C., McClafferty H., Tian L., Shipston M. J., Boguslavskyi A., Shattock M. J., and Fuller W. 2014) Substrate recognition by the cell surface palmitoyl transferase DHHC5. Proc. Natl. Acad. Sci. U.S.A. 111, 17534–17539 10.1073/pnas.1413627111 25422474 PMC4267385

[34] Percher A., Ramakrishnan S., Thinon E., Yuan X., Yount J. S., and Hang H. C. 2016) Mass-tag labeling reveals site-specific and endogenous levels of protein *S*-fatty acylation. Proc. Natl. Acad. Sci. U.S.A. 113, 4302–4307 10.1073/pnas.1602244113 27044110 PMC4843475

[35] Weber P., Batoulis H., Rink K. M., Dahlhoff S., Pinkwart K., Söllner T. H., and Lang T. 2017) Electrostatic anchoring precedes stable membrane attachment of SNAP25/SNAP23 to the plasma membrane. eLife 6, e19394 10.7554/eLife.19394 28240595 PMC5362264

[36] Gonzalo S., Greentree W. K., and Linder M. E. 1999) SNAP-25 is targeted to the plasma membrane through a novel membrane-binding domain. J. Biol. Chem. 274, 21313–21318 10.1074/jbc.274.30.21313 10409690

[37] Jiang X., Zhang Z., Cheng K., Wu Q., Jiang L., Pielak G. J., Liu M., and Li C. 2019) Membrane-mediated disorder-to-order transition of SNAP25 flexible linker facilitates its interaction with syntaxin-1 and SNARE-complex assembly. FASEB J. 33, 7985–7994 10.1096/fj.201802796R 30916996

[38] Shaaban A., Dhara M., Frisch W., Harb A., Shaib A. H., Becherer U., Bruns D., and Mohrmann R. 2019) The SNAP-25 linker supports fusion intermediates by local lipid interactions. eLife 8, e41720 10.7554/eLife.41720 30883328 PMC6422494

[39] Baghirova S., Hughes B. G., Hendzel M. J., and Schulz R. 2015) Sequential fractionation and isolation of subcellular proteins from tissue or cultured cells. MethodsX 2, 440–445 10.1016/j.mex.2015.11.001 26740924 PMC4678309

